# Role of paranasal abnormalities and systemic inflammation on primary acquired nasolacrimal duct obstruction

**DOI:** 10.1186/s12348-024-00416-y

**Published:** 2024-08-14

**Authors:** Neslihan Sevimli, Sevda Aydın Kurna, Muhammet Çakır, Sezen Akkaya

**Affiliations:** 1 Department of Ophthalmology, Fatih Sultan Mehmet Training and Research Hospital, Ataşehir, İstanbul, Turkey; 2 Department of Ophthalmology, Kızıltepe State Hospital, Mardin, Turkey; 3 Department of Ophthalmology, Dünyagöz Hospitals Group, Altunizade, İstanbul, Turkey

**Keywords:** Dacryocystorhinostomy, Lacrimal duct obstruction, Computed tomography, Inflammation

## Abstract

**Background:**

To determine the paranasal changes and inflammatory markers that may cause primary acquired nasolacrimal duct obstruction (PANDO) and to determine their relationship with success rates on different types of surgeries.

**Main body:**

We retrospectively reviewed the blood sample and computed tomography (CT) results on 92 patients who underwent dacryocystorhinostomy (DCR) surgery for PANDO and 82 healthy controls. Age, gender, paranasal abnormalities, hemogram values, International Normalized Ratio (INR) values, type of surgery, and recurrence rate were recorded; systemic Immune-inflammation Index (SII), neutrophil-to-lymphocyte ratio (NLR), monocytes-to- lymphocyte ratio (MLR) and platelet-to-lymphocyte ratio (PLR) were calculated in both groups. In the case group, total white blood cells, monocytes, and INR values were significantly lower (*p* < 0.05). Platelet, lymphocyte, neutrophil, PLR, MLR, NLR, and SII values did not differ significantly between the control and case groups (*p* > 0.05). There was no significant difference in the CT results between the groups (*p* > 0.05). No correlation was found between CT findings and inflammatory markers. Dacryocystitis (DC) was seen in 20% of patients and they were all in the case group. No correlation was found between recurrence rate and age, gender, type of surgery, CT findings, and blood results (*p* > 0.05). The recurrence rate was significantly higher in patients with bilateral PANDO and with DC (*p* < 0.05).

**Short conclusion:**

The incidence of PANDO may not be directly related to paranasal abnormalities and systemic inflammation. Low INR values may cause obstruction in the nasolacrimal duct. Age, gender, type of surgery, CT findings, and inflammation level do not affect the success of the surgery.

## Background

Nasolacrimal duct (NLD) obstruction leads to epiphora that can be managed with several surgical methods. NLD obstruction can be congenital or acquired. The acquired form can be divided into primary or secondary. Primary acquired nasolacrimal duct obstruction (PANDO) is more common in elderly females and occurs due to multifactorial factors [1, 3–9].

The most common site of NLD obstruction is in the inferior nasal meatus which has an anatomical neighborhood with the nose and paranasal sinuses [1, 3, 6, 10]. Any anatomical variation or abnormality in these structures like concha bullosa (CB), nasal septal deviation (NSD), inferior turbinate hypertrophy (ITH), mucosal thickening (MT), and the presence of a polyp or other obstruction in the nasal cavity can cause PANDO [10–12]. Also, narrow or tortuous NLD, abnormal position or shape of the lacrimal sac, and abnormalities in the bony structures surrounding the NLD can contribute to PANDO [1, 8, 13–15].

Chronic inflammation in the NLD, nasal cavity, and sinuses was accused of PANDO. This inflammation can be caused by a variety of factors, including infections, autoimmune diseases, and allergies [1, 3, 4, 10, 16, 17]. In addition, increased coagulation and fibrosis may also cause NLD obstruction [17]. Various markers like systemic immune-inflammation Index (SII) [18], neutrophil-to-lymphocyte ratio (NLR), monocytes-to-lymphocyte ratio (MLR), and platelet-to-lymphocyte ratio (PLR) can be used to identify inflammation [5, 19, 20].

Different surgical techniques can be used to treat PANDO, including external dacryocystorhinostomy (EXT-DCR), endoscopic transcanalicular diode laser dacryocystorhinostomy (LE-DCR), and mechanical endoscopic DCR (ME-DCR). Success rates can vary depending on the severity and cause of the obstruction, as well as the patient’s factors such as age, overall health, and history of prior surgery [21–24].

To achieve anatomical and functional success, determine the optimal treatment approach for PANDO, and reduce recurrences, a comprehensive evaluation that includes imaging studies such as computed tomography (CT) scans and assessment of inflammatory markers may be necessary. This can help to identify the underlying anatomical and inflammatory factors that contribute to obstruction, guide the selection of the most appropriate surgical technique, and improve surgical outcomes.

We aim to determine the anatomical differences and inflammatory markers that may cause PANDO, as well as their relationship with success rates on different types of surgery.

## Methods

This retrospective study was conducted at the Department of Ophthalmology in Fatih Sultan Mehmet Training and Research Hospital. The study was approved by the ethics committee (FSMEAH-KAEK 2022/101). The study was conducted based on the guidelines of the Declaration of Helsinki. Informed consent was obtained from the participants and was archived with the authors.

Clinical data from the patients who underwent DCR surgery between January 2015 and December 2022 due to PANDO were analyzed. The exclusion criteria were; age less than 18 years, previous nose, sinus, turbinate or lacrimal surgery, nasopharyngeal malignancy, prior history of maxillofacial fracture and NLD trauma, reflex hypersecretion, associated pathology of the lacrimal canaliculi, systemic diseases (such as cardiovascular diseases, acute/chronic kidney, diabetes, rheumatic disease), blood diseases and use of anticoagulant medication.

In total, 82 patients were included in the study. Further, 92 patients applied to the ophthalmology clinic due to blurred vision without a history of epiphora, PANDO, or DC and therefore had a blood test and CT, were included as the control group.

All studies were carried out on an Optima 660 (GE) CT with 128 rows of detectors. Patients were placed in a supine position, and the coronal and axial plane images of 1 mm thickness were obtained. Where patients had multiple scans, the latest was used. Bone structure variations and paranasal diseases such as CB, NSD, ITH, MT, existing sinusitis and DC, the presence of a polyp or other obstruction in the nasal cavity, and the osteomeatal complex (OMC) were evaluated using CT.

According to the results of blood analysis; serum white blood cell (WBC), neutrophil (N), lymphocyte (L), monocytes (M), platelet (P) values, and International Normalized Ratio (INR) values were recorded; SII, NLR, MLR, and PLR were calculated in both the case and control groups. The SII was calculated from preoperative counts of peripheral blood platelets, neutrophils, and lymphocytes per liter according to the equation (SII = P x N/L) [5].

All patients in the study group underwent a full ophthalmologic examination. Each patient’s demographic and clinical data and disease history (duration of the symptoms, recurrences, and status of complaints) were recorded.

NLD obstruction was evaluated with saline lacrimal syringing. The cannula inserted through the inferior canaliculi touched the medial wall of the lacrimal sac and a definitive endpoint known as hard stop was pushed forward. The return of saline from the lower canaliculi and not reaching the nose was considered PANDO. A total of 87 eyes, including both eyes of 5 patients, were operated with EXT-DCR (*n* = 40 eyes, 43.5%), LE-DCR (*n* = 44 eyes, 47.8%), and ME-DCR (*n* = 8 eyes, 8.7%). The choice of surgery was made according to the patient’s preference. ME-DCR was performed by an experienced otolaryngologist; EX-DCR and LE-DCR were performed by 2 experienced ophthalmologists. Subsequently, all patients underwent canalicular silicone intubation. The silicone tubes were removed 3 months postoperatively. Any epiphora symptom was recorded, the NLD patency was checked by saline lacrimal syringing and recurrences were recorded. The operation was considered “successful” for the patients who had no subjective complaints (functional success) and patent NLD (anatomical success) 1 year postoperatively.

Primary outcome measures were the anatomical and systemic factors that can cause PANDO. Secondary outcome measures were the relationship between anatomical, and systemic factors and the type of surgery and its effect on the success of the surgery.

### Statistical analysis

In the descriptive statistics of the data, mean, standard deviation, median minimum, maximum, frequency, and ratio values were used. The distribution of variables was measured with the Kolmogorov-Smirnov test. ANOVA, Independent sample t-test, Kruskal-Wallis, and Mann-Whitney u test were used in the analysis of quantitative independent data. The chi-square test was used in the analysis of qualitative independent data, and the Fischer test was used when the Chi-square test conditions were not met. SPSS 28.0 program was used in the analysis.

## Results

Our study consisted of 92 patients (30 male, 62 female) who underwent DCR surgery and 82 (30 male, 52 female) healthy control subjects. The mean ages were 56.0 ± 15.0 years in the study group and 54.4 ± 19.6 years in the control group. There was no significant difference in the age and gender ratio between groups (*p* = 0,582 and *p* = 0,143, respectively). The descriptive characteristics of the patients are presented in Table 1.

**Table 1:** Descriptive characteristics of the patients in the acquired nasolacrimal duct obstruction and control groups

		Control Group					Case Group				*p*	
		Mean ± SD/*n*-%			Median		Mean ± SD/*n*-%			Median		
Age		54.4	±	19.6	58.0		56.0	±	15.0	59.5	0.143	^t^
Gender	Female	52		63.4%			62		67.4%		0.582	^X²^
	Male	30		36.6%			30		32.6%			
WBC		7.5	±	1.8	7.7		7.0	±	1.8	6.8	**0.032**	^m^
Platelet		262.6	±	60.1	256.5		251.8	±	60.6	248.0	0.251	^t^
Lymphocyte		2.4	±	0.7	2.3		2.3	±	0.7	2.2	0.490	^t^
Monocyte		0.53	±	0.19	0.50		0.47	±	0.14	0.43	**0.044**	^m^
Neutrophil		4.4	±	1.4	4.3		4.0	±	1.3	3.9	0.078	^m^
PLR		120.9	±	42.0	110.5		119.3	±	39.1	109.4	0.933	^m^
MLR		0.24	±	0.14	0.22		0.22	±	0.09	0.21	0.329	^m^
NLR		2.1	±	1.3	1.8		1.9	±	0.8	1.8	0.696	^m^
SII		540.5	±	290.4	457.4		474.1	±	209.7	454.1	0.322	^m^
INR		1.03	±	0.09	1.01		1.00	±	0.18	0.99	**0.033**	^m^
NSD	(-)	4		16.7%			13		40.6%		0.054	^X²^
	(+)	20		83.3%			19		59.4%			
ITH	(-)	9		37.5%			14		43.8%		0.638	^X²^
	(+)	15		62.5%			18		56.3%			
MT	(-)	6		25.0%			12		37.5%		0.322	^X²^
	(+)	18		75.0%			20		62.5%			
DC	(-)	24		100.0%			20		64.5%		**0.001**	^X²^
	(+)	0		0.0%			11		35.5%			

^t^ Independent sample t-test / ^m^ Mann-whitney u test / ^X²^ Chi square test

WBC: White blood cell; PLR: platelet-to-lymphocyte ratio; MLR: monocytes-to- lymphocyte ratio; NLR: neutrophil-to-lymphocyte ratio; SII: systemic Immune-inflammation Index; INR: International Normalized Ratio; NSD: nasal septal deviation; ITH: inferior turbinate hypertrophy; MT: mucosal thickening; DC, dacryocystitis

In 48 patients (55.2%), PANDO was localized on the right side, and in 39 patients (44.8%) on the left side. Five patients (11.1%) had bilateral PANDO.

### CT scan changes in PANDO

**Fig. 1:**
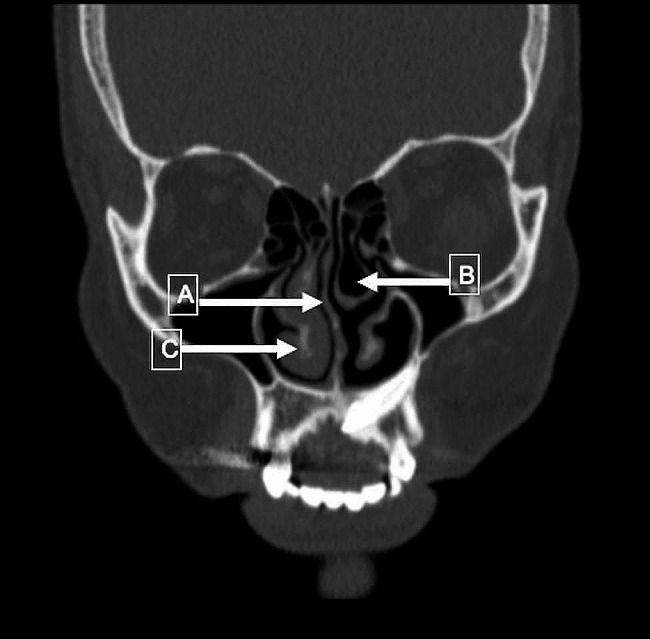
Coronal CT image of the patient who underwent external dacryocystorhinostomy for acquired nasolacrimal duct obstruction. (**A**) nasal septal deviation, (**B**) concha bullosa, (**C**) inferior turbinate hypertrophy

The most common nasal and paranasal anomalies are NSD (*n* = 39, 69.6%), ITH (*n* = 33, 58.9%), and MT (*n* = 38, 67.9%) in all groups (Fig. 1). CT scan results such as NSD, ITH, and MT ratios did not differ significantly between the control and case groups (*p* > 0.05. %). DC was seen in 20% of people (*n* = 11) and they were all in the case group (*p* = 0.001) (Table 1). No correlation was found between CT findings and inflammatory markers (Table 2).

**Table 2:** Demonstration of the relationship between tomography finding and inflammatory markers

		Computed Tomography									*p*	
		Finding-					Finding+					
		Mean ± SD/*n*-%			Median		Mean ± SD/*n*-%			Median		
WBC		7.0	±	2.2	6.9		7.1	±	1.9	6.7	0.952	^t^
Platelet		249.5	±	76.2	241.0		255.4	±	53.9	249.0	0.337	^m^
Lymphocyte		2.2	±	0.6	2.2		2.4	±	0.8	2.2	0.308	^t^
Monocyte		0.4	±	0.2	0.4		0.5	±	0.1	0.4	0.595	^t^
Neutrophil		4.2	±	1.7	4.1		4.0	±	1.3	3.9	0.687	^t^
PLR		122.1	±	36.7	107.2		117.7	±	41.7	109.4	0.836	^m^
MLR		0.2	±	0.1	0.2		0.2	±	0.1	0.2	0.985	^m^
NLR		2.0	±	1.1	1.9		1.8	±	0.7	1.7	0.446	^m^
SII		515.0	±	308.0	484.8		459.3	±	189.0	419.4	0.836	^m^
INR		1.0	±	0.1	1.0		1.0	±	0.1	1.0	0.112	^t^

^t^ Independent sample t-test / ^m^ Mann-whitney u test

WBC: White blood cell; PLR: platelet-to-lymphocyte ratio; MLR: monocytes-to- lymphocyte ratio; NLR: neutrophil-to-lymphocyte ratio; SII: systemic Immune-inflammation Index; INR: International Normalized Ratio

### Blood sample changes in PANDO

Platelet, lymphocyte, neutrophil, PLR, MLR, NLR, and SII values did not differ significantly between the control and case groups (*p* > 0.05) (Table 1).

**Fig. 2:**
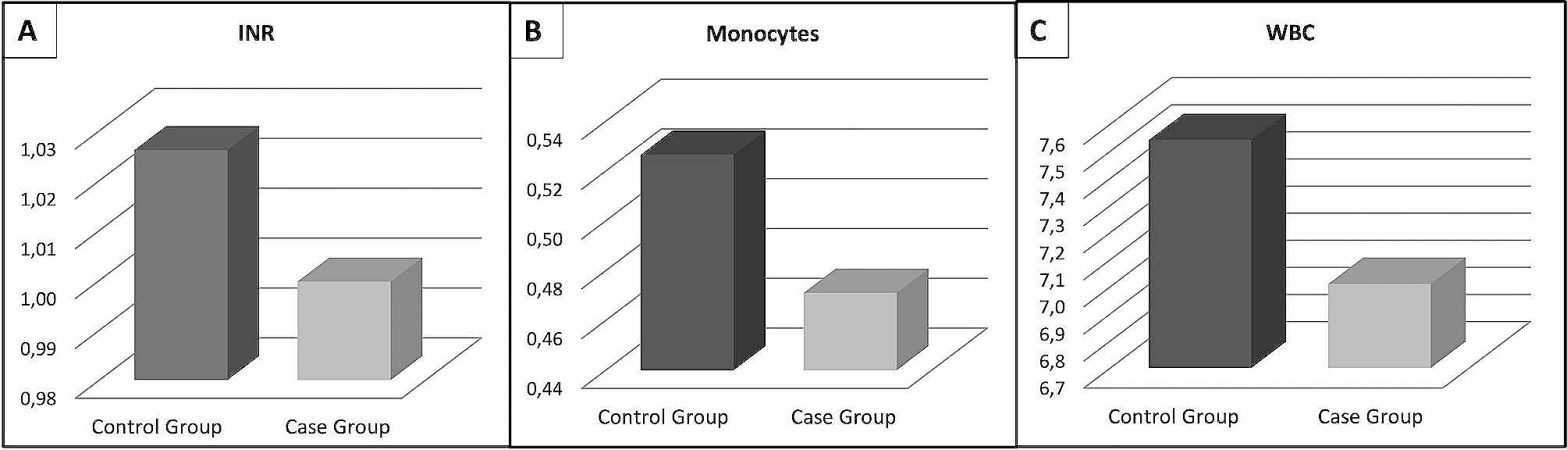
Chart of International Normalized Ratio (INR), monocytes, and white blood cell (WBC) values in case and control groups. (**A**) Change of INR values, (**B**) Change of monocyte values, (**C**) Change of WBC values in case and control groups

The mean WBC count was 7.5 ± 1.8 109/L in the control group and 7.5 ± 1.8 109/L in the PANDO group and the difference between the groups was significant (*p* = 0.032). The mean monocyte count was 0.53 ± 0.19 109/L in the control group and 0.50 ± 0.14 109/L in the PANDO group and the difference between the groups was significant (*p* = 0.044). The mean INR value was 1.03 ± 0.09 in the control group and 1.00 ± 0.18 in the PANDO group and the difference between the groups was significant (*p* = 0.033) (Table 1) (Fig. 2).

### Type of surgery

External DCR (EXT-DCR) was applied to 40 patients (43.5%), LE-DCR to 44 patients (47.8%), and ME-DCR to 8 patients (8.7%). Age and gender distribution of the patients did not differ significantly between the EXT-DCR, LE-DCR, and ME-DCR groups (*p* > 0.05). (Table 3). There was no significant difference between all surgical methods in terms of recurrence rates (*p* > 0.05) (Table 4).

**Table 3:** Demographic data of patients by type of surgery

		Type of Surgery												*p*	
			EXT-DCR				LE-DCR				ME-DCR				
Age	Mean ± SD		58.2	±	17.2		64.2	±	11.9		57.5	±	17.4	0.147	^A^
	Median		58.0				65.0				51.5				
Gender	Female	n-%	26		65.0%		30		68.2%		6		75.0%	0.849	^X²^
	Male	n-%	14		35.0%		14		31.8%		2		25.0%		
Side	Right	n-%	21		52.5%		23		52.3%		4		50.0%	0.992	^X²^
	Left	n-%	17		42.5%		18		40.9%		4		50.0%	0.892	^X²^
	Bilateral	n-%	2		5.0%		3		6.8%		0		0.0%	*p* > 0.05	^X²^

^A^ ANOVA / ^X²^ Chi square test (Fischer test)

EXT-DCR: external dacryocystorhinostomy; endoscopic transcanalicular diode laser dacryocystorhinostomy (LE-DCR) and mechanical endoscopic dacryocystorhinostomy (ME-DCR)

**Table 4:** Descriptive characteristics of the patients according to the presence of recurrence

		Recurrence (-)					Recurrence (+)				*p*	
		Mean ± SD/*n*-%			Median		Mean ± SD/*n*-%			Median		
Age		61.1	±	15.6	62.5		60.6	±	13.3	62.0	0.904	^t^
Gender	Female	47		67.1%			15		68.2%		0.928	^X²^
	Male	23		32.9%			7		31.8%			
Side	Right	40		57.1%			8		36.4%		0.089	^X²^
	Left	29		41.4%			10		45.5%		0.739	^X²^
	Bilateral	1		1.4%			4		18.2%		**0.011**	^X²^
Type of Surgery	EXT-DCR	30		42.9%			10		45.5%		0.830	^X²^
	LE-DCR	33		47.1%			11		50.0%		0.815	^X²^
	ME-DCR	7		10.0%			1		4.5%		0.675	^X²^
WBC		7.2	±	1.8	6.8		6.6	±	1.9	6.3	0.292	^m^
Platelet		253.6	±	63.1	246.5		246.0	±	52.7	249.0	0.953	^m^
Lymphocyte		2.3	±	0.7	2.2		2.2	±	0.6	2.2	0.615	^m^
Monocyte		0.5	±	0.1	0.4		0.4	±	0.1	0.4	0.379	^m^
Neutrophil		4.1	±	1.3	4.0		3.7	±	1.4	3.9	0.426	^m^
PLR		118.8	±	40.2	107.5		121.2	±	36.3	113.3	0.537	^m^
MLR		0.2	±	0.1	0.2		0.2	±	0.1	0.2	0.869	^m^
NLR		1.9	±	0.8	1.8		1.8	±	0.6	1.8	0.400	^t^
SII		483.3	±	215.0	451.8		445.1	±	193.6	468.8	0.773	^m^
INR		1.0	±	0.1	1.0		1.0	±	0.3	1.0	0.907	^m^
NSD	(-)	5		29.4%			8		53.3%		0.169	^X²^
	(+)	12		70.6%			7		46.7%			
İTH	(-)	5		29.4%			9		60.0%		0.082	^X²^
	(+)	12		70.6%			6		40.0%			
MT	(-)	4		23.5%			8		53.3%		0.082	^X²^
	(+)	13		76.5%			7		46.7%			
DC	(-)	17		100.0%			3		21.4%		**0.000**	^X²^
	(+)	0		0.0%			11		78.6%			

^t^ Independent sample t-test / ^m^ Mann-whitney u test / ^X²^ Chi square test (Fischer test)

EXT-DCR: external dacryocystorhinostomy; endoscopic transcanalicular diode laser dacryocystorhinostomy (LE-DCR) and mechanical endoscopic dacryocystorhinostomy (ME-DCR); WBC: White blood cell; PLR: platelet-to-lymphocyte ratio; MLR: monocytes-to- lymphocyte ratio; NLR: neutrophil-to-lymphocyte ratio; SII: systemic Immune-inflammation Index; INR: International Normalized Ratio; NSD: nasal septal deviation; ITH: inferior turbinate hypertrophy; MT: mucosal thickening; DC: dacryocystitis

### Recurrence rate

**Fig. 3:**
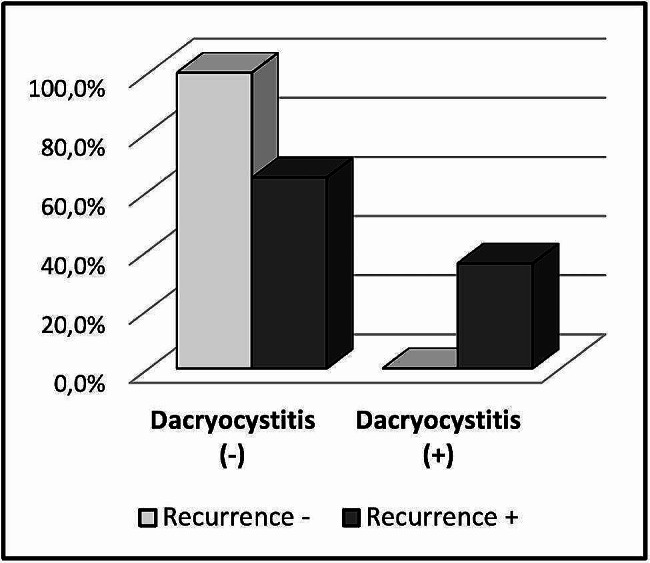
Chart of recurrence in patients who underwent dacryocystorhinostomy with and without dacryocystitis (DC)

Age and gender distribution did not differ significantly between patients with and without recurrence (*p* > 0.05). The recurrence rate did not differ significantly between the patients with normal CT results and CT results with anomaly (*p* > 0.05). There was no significant difference between all surgical methods in terms of recurrence rates (*p* > 0.05). WBC, platelet, lymphocyte, monocyte, neutrophil, PLR, MLR, NLR, S.İ.İ, INR values did not differ significantly between patients with and without recurrence (*p* > 0.05). The recurrence rate was significantly higher in patients with bilateral PANDO (*p* < 0.05). DC rate was significantly higher in patients with recurrence (*p* < 0.05) (Table 4) (Fig. 3).

## Discussion

We studied the CT images and blood samples of patients diagnosed with PANDO and undergoing surgery due to PANDO and compared them with the healthy control group in terms of the nasolacrimal system.

No significant difference was found in nasal and paranasal abnormalities like NSD, ITH, and MT in CT scans between the PANDO and the control group. DC was found to be significantly higher in the case group (*p* < 0.001). No correlation was found between CT findings and inflammatory markers.

Some studies have shown that a narrow NLD can cause PANDO and this may differ by race and gender [4, 7, 8, 12, 13, 25, 26].

Some studies are showing that a narrow-angle between the bone of the inferior turbinate and the upper part of the medial wall of the maxillary sinus might be a possible causative factor in the PANDO [1, 10, 15]. A study demonstrated that patients with chronic PANDO can have characteristic bone changes [14].

Lin et al. showed that people with PANDO have special demographic and facial characteristics as it appears less in men due to facial thickening. In our study, the majority of those with PANDO were female [4].

In contrast to our study, some studies demonstrate a correlation between CT findings of sinus disease or nasal abnormality because of the lacrimal system’s anatomic proximity to the lateral wall of the nose [6, 11, 27]. While Habesoglu et al. [11] found a significant increase in the rate of CB, ITH, OMC disease, and maxillary sinusitis, Kallman et al. [6] found that ethmoidal opacification, agger nasi cell opacification, and NSD rates were significantly higher in PANDO patients.

Similar to our study, there are also studies showing that there is no relationship between nasal anomalies and paranasal diseases, and PANDO [22, 24–28]. Kule et al. found no statistically significant difference for the NSD, agger nasi, CB, OMC, or ethmoid pathologies between the PANDO and control groups [28].

Many studies have shown that chronic inflammation causes PANDO [7, 13, 16, 26, 29]. Makselis et al. [16] showed chronic non-granulomatous inflammation of the lacrimal sac histologically. For this purpose, we tried to determine the inflammation markers that may cause PANDO by examining the hemogram values of PANDO patients in our study. We did not find any significant difference between the groups in NLR, MLR, PLR, and SII values, which are used as indicators of systemic inflammation (*p* > 0.05). Furthermore, we found no correlation between CT findings and inflammatory markers. Atum et al. [5] found the NLR values to be significantly higher, while the mean platelet volume values were significantly lower in the PANDO group. Our study is the first study in the literature to evaluate SII and INR values in PANDO patients to our knowledge. We found INR values significantly lower in the case group (*p* < 0.05). The significantly lower INR value in our study suggests that increased coagulation may cause PANDO. Monocytes are known to play an essential role in inflammation, on the contrary, we found the monocyte values to be significantly lower in the PANDO group [30]. Accordingly, we believe WBC values may have been significantly lower.

The secondary outcome was DCR success, defined as regression of symptoms and/or patent NLD on saline lacrimal syringing. We did not find a significant difference in functional and anatomical success results between the surgical methods we applied. Although a smaller opening between the lacrimal sac and nasal cavity causes a higher recurrence rate in LE-DCR, it may be preferred for shorter operative time, while reducing the chance of complications such as bleeding or wound infection and cosmetic results [21–24]. We chose the surgical method according to the patient’s preference.

Huang et al. found that EXT-DCR had slightly better success rates than LE-DCR and ME-DCR in their meta-analysis [21]. LE-DCR had poorer outcomes in many studies [21–23]. Yılmaz et al. [22] found similar success and complication rates with 3 DCR methods in patients with PANDO. We intubated all our patients with a silicone tube to be removed after 3 months. In a study, DCR with intubation achieved better results than DCR without intubation [31].

In our study, the recurrence rate was found to be irrelevant in terms of age, sex, CT findings, and inflammatory markers. Recurrences were more common in those with only bilateral and DC.

The limitation of this study is that it is retrospective. Only recent blood values were considered. No bone changes or NLD length and width measurements were made and changes in the lacrimal microenvironment that may develop due to inflammation could not be demonstrated. In addition, there was no long follow-up period. We chose the surgical method according to the patient’s preference. We could choose the ME_DCR method for those with severe bone stenosis, which involves creating a new opening in the bone to bypass the obstruction. Similarly, patients with evidence of chronic inflammation may require more aggressive medical management to control their symptoms and improve surgical outcomes. This may involve the use of topical or systemic steroids, antibiotics, or immunomodulatory agents. The ME-DCR group was a much smaller group than the others. The surgeries were performed by three different surgeons from two different branches.

## Conclusion

PANDO is a common condition that can have a significant impact on a patient’s quality of life. We could not determine the relationship of inflammatory markers or CT images with PANDO in our study. Even if there is no reason to determine the optimal treatment approach for PANDO, a comprehensive evaluation that includes imaging studies and assessment of inflammatory markers may be necessary. The success rates of different surgical procedures may depend on the severity of the obstruction, the underlying anatomical abnormalities, and the degree of inflammation.

According to our research, there is no previous article investigating a relationship between INR values and PANDO. In addition to the fact that low INR can cause PANDO, excessive bleeding may occur during surgery and cause complications in patients using blood thinners if they have high INR values. The INR level may need to be closely monitored and adjusted accordingly to ensure safe and effective treatment.

## Data Availability

The data presented in this study are available on request from the corresponding author.

## Abbreviations

CB: Concha bullosa
CT: Computed tomography
DC: Dacryocystitis
DCR: Dacryocystorhinostomy
EXT-DCR: External dacryocystorhinostomy
INR: International Normalized Ratio
ITH: Inferior turbinate hypertrophy
L: Lymphocytes
LE-DCR: Endoscopic transcanalicular diode laser dacryocystorhinostomy
ME-DCR: Mechanical endoscopic DCR
MLR: Monocytes-to- lymphocyte ratio
MT: Mucosal thickening
N: Neutrophil
NLD: Nasolacrimal duct
NLR: Neutrophil-to-lymphocyte ratio
NSD: Nasal septal deviation
OMC: Osteomeatal complex
P: Platelets
PANDO: Primary acquired nasolacrimal duct obstruction
PLR: Platelet-to-lymphocyte ratio
SII: Systemic Immune-inflammation Index
WBC: Serum white blood cell
